# Usefulness of continuous glucose monitoring of blood glucose control in patients with diabetes undergoing hemodialysis: A pilot study

**DOI:** 10.3389/fmed.2023.1145470

**Published:** 2023-04-06

**Authors:** Sua Lee, Soyoung Lee, Kyeong Min Kim, Jong Ho Shin

**Affiliations:** Division of Nephrology, Department of Internal Medicine, Daejeon Eulji Medical Center, Eulji University School of Medicine, Daejeon, South Korea

**Keywords:** continuous glucose monitoring, glucose control, glycemic variability, hemodialysis, uncontrolled diabetes

## Abstract

**Background:**

Blood glucose stability has recently been considered important in the treatment of diabetes. Both hypoglycemia and hyperglycemia can frequently occur in patients with diabetes undergoing hemodialysis. This study aimed to determine the usefulness of continuous glucose monitoring (CGM) for glycemic control and glycemic variability stabilization in patients with diabetes undergoing hemodialysis.

**Materials and methods:**

Eighteen patients aged ≥18 years with type 1 or 2 diabetes and ≥3 months on hemodialysis at the Eulji Medical Center, Daejeon, Republic of Korea between November 2021 and May 2022 were included. Patients underwent **7** days CGM twice: the baseline study period (T0) and the follow-up study period (T1), at a 12 weeks interval. Physicians modified the treatment strategy according to the T0 results, and then patients conducted T1. As indicators of glycemic control, the mean glucose levels, glycated hemoglobin A1c (HbA1c), and time in range were measured. As indicators of glycemic variability, standard deviation (SD) and % coefficient variation (%CV) were measured.

**Results:**

Data from 18 patients were analyzed. The mean glucose levels, HbA1c, SD, and %CV improved in T1 compared to T0 (*P* < 0.05). During T0, the mean glucose level was significantly lower on a day with hemodialysis than on a day without (*P* < 0.05), and SD and %CV were significantly higher on a day with hemodialysis than on a day without (*P* < 0.05). After the physicians modified the treatment according to the T0 results, there were no differences in the mean glucose levels, SD, and %CV between days with and without hemodialysis during T1.

**Conclusion:**

Continuous glucose monitoring could be a promising tool for individualizing treatment strategies in patients with diabetes undergoing hemodialysis.

## 1. Introduction

End-stage kidney disease (ESKD) is a global health problem, with a steep increase in its prevalence (1). The prevalence and incidence of ESKD are also steadily increasing in South Korea (2, 3). Diabetes is a major cause of ESKD and a common comorbidity in patients with chronic kidney disease (1, 3). Patients with ESKD have a high prevalence of cardiovascular complications and high mortality rate, and the risk of cardiovascular morbidity and mortality is even higher in patients with both diabetes and ESKD (3–5).

Glycemic control is an important factor in preventing micro- and macrovascular complications in patients with diabetes. However, it is particularly challenging in patients with diabetes undergoing hemodialysis because the kidney is an organ closely related to blood glucose and insulin metabolism, and glucose and insulin are filtered through the dialysis membrane (6, 7). Therefore, both hypoglycemia and hyperglycemia may frequently occur in patients with diabetes undergoing hemodialysis. Moreover, the recommendation for strict glycemic control in patients with diabetes undergoing hemodialysis is controversial because of the increased risk of hypoglycemia (8, 9).

Self-monitoring capillary blood glucose (SMBG), glycated hemoglobin A1c (HbA1c), and fructosamine are commonly used in patients with diabetes to monitor glycemic control (10). However, HbA1c and fructosamine have limitations as standard indicators of glycemic control in patients with diabetes undergoing hemodialysis for several reasons, such as anemia, hypoalbuminemia, and analytical interference (10, 11). In addition, the HbA1c and fructosamine levels cannot reflect glycemic variability, which is usually defined as the frequency and amplitude of blood glucose oscillations, or changes in acute blood glucose (12). Glycemic variability has been suggested to be an independent risk factor for morbidity and mortality in patients with diabetes (13). The American Diabetes Association 2022 Standards of Medical Care in Diabetes suggested the stabilization of glycemic variability as one of the treatment goals in diabetes and the use of continuous glucose monitoring (CGM) as a surrogate tool for glucose assessment (14). CGM is a Holter-like device to monitor interstitial glucose, which detects several glycemic parameters such as mean, peak, nadir, pattern, and variability (15). Therefore, interest in CGM has increased in recent years. We aimed to examine the blood glucose levels in patients with diabetes undergoing hemodialysis using CGM and improve their blood glucose levels and achieve stabilization of glycemic variability through individualized treatment interventions based on the CGM results.

## 2. Materials and methods

### 2.1. Participants and data sources

This study was approved by the Ethics Committee of Eulji University School of Medicine in South Korea (IRB No. EMC 2021-07-013-001) and was conducted in accordance with the principles of the Declaration of Helsinki. All study participants provided written informed consent prior to enrollment in the study.

This prospective cohort study included patients with ESKD aged ≥18 years who received standard hemodialysis in the dialysis center at Daejeon Eulji Medical Center between November 2021 and May 2022. The inclusion criteria were as follows: (a) having type 1 or type 2 diabetes for ≥3 months, (b) having undergone any combination of diabetes treatment for ≥3 months, (c) maintained stable hemodialysis for ≥3 months, and (d) undergoing hemodialysis for 4 h three times a week. Patients were excluded if they (a) were pregnant, (b) had a concurrent systemic inflammatory disease or acute infection, (c) had been hospitalized because of diabetic ketoacidosis or hyperglycemic hyperosmolar syndrome within the last 3 months, (d) had received blood transfusion within the last 3 months, (e) had a history of cerebral vascular accident and angina pectoris or acute myocardial infarction within the last 3 months, (f) had insufficient life expectancy due to malignancy or other medical conditions, or (g) did not understand the study protocol or agree to participate in this study. Clinical data, such as age, sex, body mass index (BMI), comorbid disease, used diabetes medication, use of erythropoiesis-stimulating agent (ESA), and dose of ESA, were collected from the patients’ electronic medical records.

### 2.2. Study protocol

**FIGURE 1:**
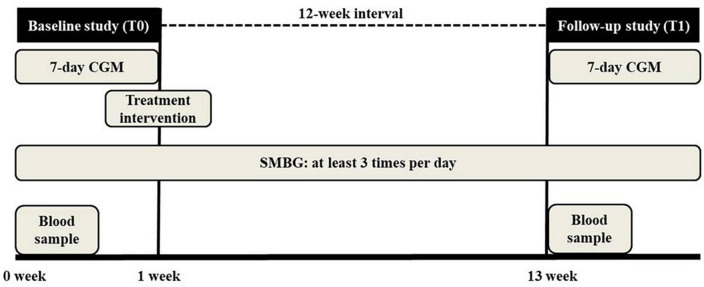
Study protocol. Patients underwent 7 days continuous glucose monitoring (CGM) twice at a 12 weeks interval. In the baseline study period (T0), physicians and patients measured the basal glucose levels and glycemic markers without intervention. Physicians modified the treatment strategy based on the T0 results. After 12 weeks, the follow-up study (T1) was conducted. Patients checked self-monitoring capillary blood glucose (SMBG) at least three times per day during the study.

This study utilized two CGM study periods. In the baseline study period (T0), physicians and patients measured the basal glucose levels and glycemic markers without intervention, such as control of diet, exercise, or medication modification. The CGM device was inserted on the hemodialysis-on day, after dialysis, and was maintained for 7 days, after which it was removed on the last day. During the study period, patients were asked to measure SMBG at least three times per day and record the type, amount, and time of all meals, including snacks, medications, exercise, and episodes of hypoglycemia in the diary given by the physician. Based on the results of T0, patients whose glycemic markers were outside of the target range were educated on exercise and dietary habits, and treatment changes were administered. After 12 weeks of treatment, the follow-up study period (T1) commenced in the same manner as for T0. The study protocol is presented in Figure 1.

### 2.3. Treatment intervention

The two nephrologists were asked to change the prescription based on the CGM report while blinding the patient’s name. A comprehensive treatment regimen could not be applied due to the complexity of dialysis patients with diabetes. For patients with overall hyperglycemia, with or without dialysis, the doses of medication were increased by 25–50% or insulin doses by 2–6 units. On the other hand, in patients with frequent hypoglycemia, with or without dialysis, the doses of medication were reduced by 25–50% or insulin doses by 2–6 units. In patients with recurrent episodes of hypoglycemia during dialysis, it was recommended to avoid medication or insulin administration on the morning of the dialysis day, and to administer the medication or insulin immediately before a meal after dialysis. Patients who did not have hypoglycemia during dialysis but who developed hyperglycemia after dialysis were instructed to take additional medication or administer additional insulin after dialysis. The treatment interventions for each of all patients are shown in Supplementary Table 1.

### 2.4. CGM

iPro2 CGM (MMT-7745) and Enlite glucose sensors MMT-7008A (Medtronic Minimed, Northridge, CA, USA) were used to assess glycemic control. The enzyme-driven oxidation of glucose in the interstitial fluid generates a current that is recorded every 10 s and is stored every 5 min by the device. CGM was performed for seven consecutive days. The device was applied to the subcutaneous tissue of the anterior abdominal wall. Patients were asked to undergo SMBG at least three times per day during the CGM period for retrospective calibration. As the patients were blinded to real-time glucose levels, the CGM system was able to prevent the patients’ behavior from affecting the CGM results. The accuracy of the CGM device compared with that of the serum glucose levels had already been reported in several previous studies (10, 16).

### 2.5. Assessment of CGM metrics

Continuous glucose monitoring metrics were defined and assessed according to the International Consensus of the Advanced Technologies & Treatments for Diabetes Congress in February 2019 (17), as follows.

i) Mean glucose: CGM measured every 10 s and recorded every 5 min.
ii) Time in range (TIR): % of readings and time within the target glucose range of 70–180 mg/dL.
iii) Time below range (TBR): % of readings and time below a blood glucose level of 70 mg/dL.
iv) Time above range (TAR): % of readings and time above a blood glucose level of 180 mg/dL.
v) Over-area under the curve (AUC) above 180 mg/dL: The AUC for glucose levels measured above 180 mg/dL by CGM was assessed.
vi) Standard deviation (SD): one of the indicators of glycemic variability. SD is a measure of the spread of glucose readings around the average.
vii) % Coefficient of variation (%CV): one of the indicators of glycemic variability. %CV was obtained by dividing SD by the mean glucose and multiplying by 100 to obtain a percentage.

### 2.6. Glycated hemoglobin A1c

Blood samples were obtained at the initiation of a dialysis session on the day preceding CGM for each study period. HbA1c levels were measured using high-performance liquid chromatography. Since HbA1c reflects the average blood glucose levels for 2–3 months, the study conducted at 12 weeks intervals to determine the improvement of average blood glucose levels without overlap between T0 and T1.

### 2.7. Laboratory data

Blood samples were obtained at the start of a dialysis session on the day preceding CGM during T0. The hemoglobin, hematocrit, blood urea nitrogen, serum creatinine, serum total protein, serum albumin, high-sensitivity C-reactive protein, serum iron, serum ferritin, total iron binding capacity, and transferrin saturation levels were measured. Renal function was assessed by modification of diet in renal disease-estimated glomerular filtration rate (MDRD-eGFR). The Kt/V was obtained as a value automatically calculated by the electronic medical system.

### 2.8. Statistical analysis

Continuous variables are presented as means ± SDs. Categorical variables are expressed as frequencies and percentages. The paired *t*-test was used to compare the glycemic markers of the baseline and follow-up studies with a normal distribution. The Wilcoxon signed-rank test was used to compare the glycemic markers of the baseline and follow-up studies with a non-normal distribution. A *P*-value < 0.05 was considered to be the limit of statistical significance. All statistical analyses were performed using Statistical Package for the Social Sciences software version 24 (IBM Inc., Armonk, NY, USA).

## 3. Results

### 3.1. Basic demographics and clinical characteristics

Twenty patients were enrolled in this study, but two patients dropped out between T0 and T1 due to acute inflammatory disease and gastrointestinal bleeding. Thus, 18 patients were analyzed. The basic demographic and clinical characteristics of the patients are presented in Table 1. The mean age was 62.0 ± 11.2 years, male/female ratio was 13 (72.2%)/5 (27.8%), and mean BMI was 23.9 ± 3.3 kg/m^2^. Hypertension was the most common comorbidity in this study (16 patients, 88.9%). Eight (44.4%) and two (11.1%) patients had a history of coronary artery disease and cerebral vascular accidents, respectively. The mean dialysis duration was 5.2 ± 3.5 years. Among the 18 patients, only one had type 1 diabetes, and the mean diabetes duration was 22.9 ± 7.0 years. Ten patients (55.6%) were on insulin therapy, including multiple subcutaneous insulin injections (*n* = 5), premixed insulin (*n* = 2), premixed insulin plus oral antidiabetic agents (OAD) (*n* = 2), or basal insulin plus OAD (*n* = 1). Eight patients (44.4%) were on OAD alone. Sixteen patients (88.9%) were on ESA for the treatment of anemia, and eight patients each were on erythropoietin (EPO) and darbepoetin alfa. The mean EPO dose of ESA was 6781.3 ± 5689.1 IU/week.

**TABLE 1:** Basic demographics and clinical characteristics.

Variables	Patients (*n* = 18)
Age at diagnosis (years), mean ± SD	62.0 ± 11.2
Sex, male/female, *n* (%)	13 (72.2)/5 (27.8)
BMI (kg/m^2^), mean ± SD	23.9 ± 3.3
**Comorbid diseases, *n* (%)**	
HTN	16 (88.9)
CAD	8 (44.4)
CVA	2 (11.1)
Dialysis duration (years), mean ± SD	5.2 ± 3.5
Type of diabetes mellitus, Type 1/Type 2, n (%)	1 (5.6)/17 (94.4)
Diabetes duration (years), mean ± SD	22.9 ± 7.0
**Diabetes medication, *n* (%)**	
OAD	8 (44.4)
MSII	5 (27.8)
Premix insulin	2 (11.1)
Premix insulin + OAD	2 (11.1)
Basal insulin + OAD	1 (5.5)
**Use of ESA, *n* (%)**	
EPO	8 (40)
DA	8 (40)
Dose of ESA, as EPO (IU/week)	6781.3 ± 5689.1
**Laboratory findings**	
Hb at T0/T1 (g/dL), mean ± SD	10.2 ± 0.9/10.9 ± 1.1
Hct at T0/T1 (%), mean ± SD	31.4 ± 2.4/33.5 ± 3.2
BUN (mg/dL), mean ± SD	69.1 ± 21.0
Cr (mg/dL), mean ± SD	10.0 ± 2.4
MDRD-eGFR (mL/min/1.73 m^2^), mean ± SD	5.2 ± 1.2
Serum total protein at T0/T1 (g/dL), mean ± SD	6.9 ± 0.7/6.9 ± 0.8
Serum albumin at T0/T1 (g/dL), mean ± SD	4.1 ± 0.3/4.1 ± 0.3
hs-CRP at T0/T1 (mg/dL), mean ± SD	0.7 ± 1.4/0.3 ± 0.2
Serum iron (μg/dL), mean ± SD	52.8 ± 17.7
Serum ferritin (ng/dL), mean ± SD	270.1 ± 206.6
TIBC (μg/dL), mean ± SD	253.2 ± 48.6
TSAT (%), mean ± SD	21.8 ± 9.2
Kt/V at T0/T1, mean ± SD	1.41 ± 0.18/1.45 ± 0.16

SD, standard deviation; BMI, body mass index; HTN, hypertension; CAD, cardiovascular disease; CVA, cerebrovascular accident; OAD, oral antidiabetic agents; MSII, multiple subcutaneous insulin injection; ESA, erythropoiesis stimulating agent; EPO, erythropoietin; DA, darbepoetin alfa; IU, international unit; Hb, hemoglobin; Hct, hematocrit; BUN, blood urea nitrogen; Cr, creatinine; MDRD-eGFR, modification of diet in renal disease estimated glomerular filtration rate; hs-CRP, high-sensitivity C-reactive protein; TIBC, total iron binding capacity; TSAT, transferrin saturation.

Based on the laboratory findings, the mean hemoglobin and hematocrit levels were 10.2 ± 0.9 g/dL and 31.4 ± 2.4%, respectively. Regarding renal function, the mean serum creatinine levels and MDRD-eGFR were 10.0 ± 2.4 mg/dL and 5.2 ± 1.2 mL/min/1.73 m^2^, respectively. The mean serum total protein and albumin levels were 6.9 ± 0.7 g/dL and 4.1 ± 0.3 g/dL, respectively, and the mean Kt/V was 1.41 ± 0.18.

All patients underwent hemodialysis with a dialysate of the same glucose concentration (175 mg/dL) because of our center’s central dialysis fluid delivery system.

### 3.2. Changes in glycemic markers and CGM metrics

**FIGURE 2:**
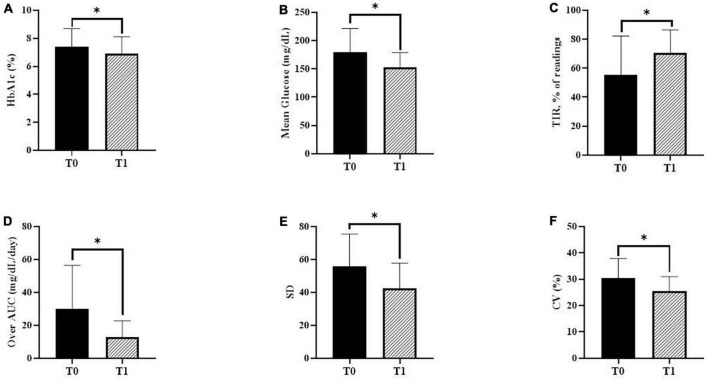
Changes in glycemic markers and continuous glucose monitoring (CGM) metrics. **(A)** Glycated hemoglobin A1c (HbA1c) during baseline (T0) and follow-up (T1) (*P* = 0.023). **(B)** The mean glucose levels using CGM during T0 and T1 (*P* = 0.001). **(C)** The mean percentage of readings within time in range (TIR) during T0 and T1 (*P* < 0.001). **(D)** The over-area under the curve (AUC) during T0 and T1 (*P* = 0.001). **(E)** The standard deviation (SD) during T0 and T1 (*P* = 0.001). **(F)** Percentage coefficient of variation (%CV) during T0 and T1 (*P* < 0.001). *P*-values were calculated using the paired *t*-test or the Wilcoxon signed-rank test. *P < 0.05 was considered statistically significant.

Changes in glycemic markers and CGM metrics are presented in Figure 2. The mean HbA1c value decreased statistically significantly from 7.4 ± 1.3% during T0 to 6.9 ± 1.2% during T1 (*P* = 0.023). Regarding CGM metrics, the mean blood glucose levels decreased statistically significantly from 179.1 ± 42.3 mg/dL during T0 to 153.2 ± 25.6 mg/dL during T1 (*P* = 0.001). The mean percentage of readings and time per day in TIR significantly increased (55.6 ± 26.7 and 11.2 ± 6.6 during T0 vs. 70.5 ± 15.9% and 14.6 ± 4.4 h during T1; *P* < 0.001). In addition, the Over-AUC significantly decreased from 29.9 ± 26.5 mg/dL/day during T0 to 12.7 ± 10.1 mg/dL/day during T1 (*P* = 0.001). Regarding glycemic variability indicators, SD significantly decreased from 55.7 ± 19.8 mg/dL during T0 to 42.6 ± 15.3 mg/dL during T1 (*P* = 0.001). %CV significantly decreased from 30.5 ± 7.3% during T0 to 25.5 ± 5.5% during T1 (*P* < 0.001). The TBR and AUC values below 70 mg/dL were very low, with no statistically significant changes between T0 and T1.

### 3.3. Changes in CGM metrics according to the day with or without hemodialysis sessions

**FIGURE 3:**
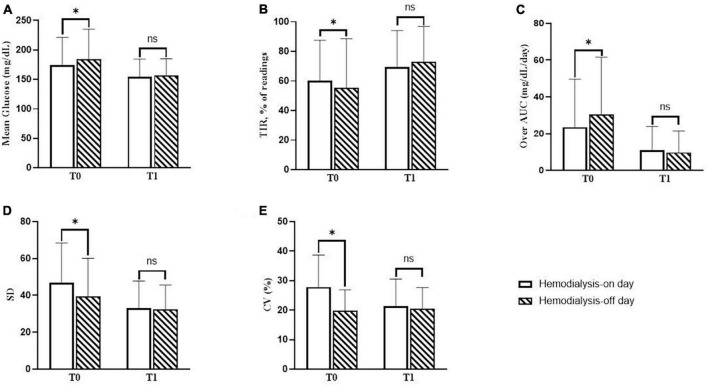
Changes in continuous glucose monitoring (CGM) metrics on days with or without hemodialysis sessions. **(A)** The difference in mean glucose levels between the hemodialysis-on and hemodialysis-off days during baseline (T0) and follow-up (T1). **(B)** The difference in mean percentage of readings within time in range (TIR) between the hemodialysis-on and hemodialysis-off days during T0 and T1. **(C)** The difference in over-area under the curve (AUC) between the hemodialysis-on and hemodialysis-off days during T0 and T1. **(D)** The difference in standard deviation (SD) between the hemodialysis-on and hemodialysis-off days during T0 and T1. **(E)** The difference in percentage the coefficient of variation (%CV) between the hemodialysis-on and hemodialysis-off days during T0 and T1. *P*-values were calculated using the paired *t*-test or the Wilcoxon signed-rank test. *P < 0.05 was considered statistically significant.

For each patient, CGM metrics were assessed for at least 2 days each on the hemodialysis-on day and the hemodialysis-off day. The changes in the CGM metrics according to the day with or without hemodialysis sessions are presented in Figure 3. The blood glucose levels from 1 h before dialysis to 4 h after dialysis on a hemodialysis-on day were compared to those during the same time period on a hemodialysis-off day.

During T0, the mean blood glucose level on the hemodialysis-on day was statistically significantly lower than that on the hemodialysis-off day (174.7 ± 46.5 vs. 184.7 ± 50.5 mg/dL, *P* = 0.001). The mean percentage of readings and time per day in TIR on the hemodialysis-on day were all statistically significantly higher than those on the hemodialysis-off day (60.3 ± 27.2 and 14.0 ± 6.8 vs. 55.4 ± 33.2% and 13.2 ± 7.8 h, *P* = 0.016). The Over-AUC on the hemodialysis-on day was statistically significantly lower than that on the hemodialysis-off day (23.5 ± 26.1 vs. 30.3 ± 31.3 mg/dL/day, *P* = 0.001). Regarding glycemic variability indicators, SD on the hemodialysis-on day was statistically significantly higher than that on the hemodialysis-off day (46.9 ± 21.5 vs. 39.5 ± 20.6 mg/dL, *P* = 0.007). %CV on the hemodialysis-on day was statistically significantly higher than that on the hemodialysis-off day (27.8 ± 10.9 vs. 19.7 ± 7.2%, *P* = 0.001).

During T1, the mean blood glucose levels significantly decreased on both the hemodialysis-on and hemodialysis-off days, as compared to those values during T0 (174.7 ± 46.5 vs. 154.6 ± 29.7 mg/dL, *P* = 0.001; 184.7 ± 50.5 vs. 156.3 ± 28.7 mg/dL, *P* < 0.001, respectively), and consequently there were no statistically significant differences between the hemodialysis-on and hemodialysis-off days during T1 (*P* = 0.638). The mean percentages of readings in TIR significantly increased on both the hemodialysis-on and hemodialysis-off days, as compared to those values during at T0 (60.3 ± 27.2 vs. 69.3 ± 24.8%, *P* = 0.016; 55.4 ± 33.2 vs. 73.1 ± 23.8%, *P* < 0.001, respectively), and consequently there were no statistically significant differences between the hemodialysis-on and hemodialysis-off days during T1 (*P* = 0.511). The Over-AUC significantly decreased on both the hemodialysis-on and hemodialysis-off days compared to those values during T0 (25.3 ± 26.1 vs. 11.1 ± 12.8 mg/dL/day, *P* = 0.001; 30.3 ± 31.3 vs. 9.8 ± 11.6 mg/dL/day, *P* < 0.001, respectively), and consequently there were no statistically significant differences between the hemodialysis-on and hemodialysis-off days during T1 (*P* = 0.637). Regarding indicators of glycemic variability, SD was statistically significantly decreased on both the hemodialysis-on and hemodialysis-off days compared to those values during T0 (46.9 ± 21.5 vs. 33.2 ± 14.6, *P* = 0.009; 39.5 ± 20.6 vs. 32.3 ± 13.3, *P* = 0.002, respectively), and consequently, there were no statistically significant differences between the hemodialysis-on and hemodialysis-off days during T1 (*P* = 0.384). %CV significantly decreased on the hemodialysis-on day during T1 compared to that during T0 (27.8 ± 10.9 vs. 21.4 ± 9.2, *P* = 0.014), and there was no statistically significant difference between the hemodialysis-on and hemodialysis-off days during T1 (*P* = 0.166).

**FIGURE 4:**
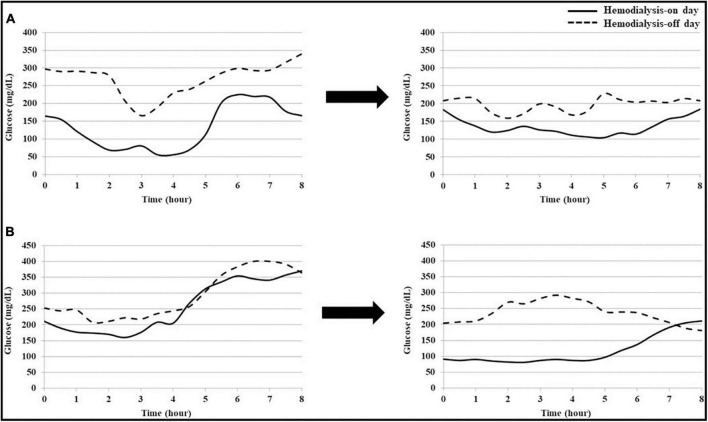
Changes in blood glucose patterns of each patient with hypoglycemia or hyperglycemia. **(A)** Patient 1 frequently had hypoglycemia, with blood glucose levels below 70 mg/dL, during dialysis. After treatment intervention using continuous glucose monitoring (CGM), hypoglycemia during dialysis was improved. **(B)** Patient 2 frequently had hyperglycemia, with a blood glucose level above 250 mg/dL, regardless of dialysis. After treatment intervention using CGM, hyperglycemia was improved on the hemodialysis-on and off days.

The changes in blood glucose patterns in patients with frequent hypoglycemia (Patient 1) and frequent hyperglycemia (Patient 2) are presented in Figure 4. Patients 1 had frequent episodes of hypoglycemia during dialysis and Patient 2 developed persistent hyperglycemia, respectively. Both patients showed high glycemic variability and did not meet the recommended TIR values (Patient 1; SD 80 mg/dL, %CV 35%, and TIR 32%, Patient 2; SD 70 mg/dL, %CV 31%, and TIR 11%). During T1, hypoglycemia during the dialysis session was improved in Patient 1, and the blood glucose level decreased on the hemodialysis-on and off days in Patient 2. In both patients, the percentage of readings and time per day within the TIR increased, and glycemic variability was improved (Patient 1; SD 36 mg/dL, %CV 19%, and TIR 52%, Patient 2; SD 51 mg/dL, %CV 21%, and TIR 52%).

## 4. Discussion

Continuous glucose monitoring is reportedly more useful as a blood glucose monitoring or assessment tool than SMBG, HbA1c, and fructosamine in patients with diabetes undergoing hemodialysis (5, 15, 18–20). Our study indicated that CGM could be used to improve the blood glucose levels and monitor or assess blood glucose. First, through the treatment intervention based on CGM metrics, both the mean glucose and HbA1c levels were significantly decreased, indicating overall improvement in the blood glucose levels. Treatment intervention was performed individually for each patient. Patients who had hypoglycemia on the day of hemodialysis on CGM results were advised to take OAD or insulin after an afternoon meal. Patients with hyperglycemia regardless of hemodialysis were prescribed increased OAD or insulin. In the diary record, patients who frequently ate snacks or late-night meals were given individual education on the importance of glycemic control and were instructed to reduce snacks and late-night meals. A strong correlation of SMBG with the blood glucose levels in CGM has already been reported in previous studies, but findings on the correlation between the HbA1c and blood glucose levels in CGM among patients undergoing hemodialysis were heterogeneous across studies (20–23). In the present study, treatment intervention based on CGM metrics made it possible to improve the mean glucose and HbA1c levels. Comparing CGM metrics at baseline with those after treatment intervention, the mean percentage of readings and time per day within TIR increased and Over-AUC decreased. Although no standard target for CGM metrics has been established to date, the mean percentage of TIR over 70% is the recommended goal suggested by international consensus (17). The value of TIR 70% has been reported to correspond to an HbA1c of approximately 6.7 or 7% (24, 25). In our study, HbA1c after treatment intervention, based on CGM metrics, was 6.9 ± 1.2%, consistent with those findings. Patients included in the present study tended to have hyperglycemia and experienced an improvement in hyperglycemia with CGM-based treatment intervention. Improving hyperglycemia is important to delay the progression of micro- and macrovascular complications and improve the survival rate in patients with diabetes (26, 27).

In the present study, both SD and %CV as indicators of glycemic variability decreased after CGM-based treatment intervention. Several studies have reported the effects of glycemic variability on various diseases, such as nephropathy, retinopathy, neuropathy, diabetic foot ulcer, chronic obstructive pulmonary disease, and Alzheimer’s disease (4, 28–32). In addition, the correlation between glycemic variability and markers of vascular endothelial dysfunction, such as baroreflex sensitivity, excessive glycation, and the generation of oxidative stress, has been previously reported (33, 34). Recent systematic reviews have suggested that glycemic variability may be an independent risk factor for micro- and macrovascular complications and mortality (13, 19).

We identified differences in the blood glucose patterns according to hemodialysis. In the baseline study period, the mean glucose levels on the hemodialysis-on day were significantly lower than those on the hemodialysis-off day. However, both SD and %CV, as glycemic variability indicators, were higher on the hemodialysis-on day than on the hemodialysis-off day. Two patients (11.1%) had asymptomatic hypoglycemia during hemodialysis and another two patients (11.1%) had asymptomatic hypoglycemia within 2 h after the end of hemodialysis. Several previous studies have reported that the mean blood glucose levels were significantly lower on the hemodialysis-on day than on the hemodialysis-off day, and the frequency of hypoglycemia was higher on the hemodialysis-on day than on the hemodialysis-off day (6, 15, 22, 35). Because of this phenomenon, glycemic treatment to prevent hypoglycemia during dialysis was administered to most patients before enrollment in the present study. Therefore, the mean glucose levels and the AUC were significantly higher on the hemodialysis-off day than on the hemodialysis-on day during the baseline period. We speculate that several factors, such as intradialytic hypoglycemia, treatment for preventing intradialytic hypoglycemia, and hyperglycemia on the hemodialysis-off day, could lead to poor glycemic control and glycemic variability in patients with diabetes undergoing hemodialysis. In the present study, it was observed that the overall blood glucose levels and glycemic variability were improved by analyzing blood glucose patterns according to dialysis for each patient and individualizing treatment based on CGM metrics.

In patients with ESKD, glucose metabolism and increased insulin resistance are altered by multifactorial mechanisms, including chronic inflammation, oxidative stress, vitamin D deficiency, metabolic acidosis, anemia, decreased glucose uptake in the liver and muscle, increased gluconeogenesis and lipogenesis in the liver, and decreased glycogen and protein synthesis in the muscle (36). In addition, renal dysfunction leads to changes in the pharmacokinetics of exogenous insulin and oral antidiabetic agents, resulting in increased frequency of hypo- and hyperglycemia. In patients undergoing hemodialysis, blood glucose is rapidly removed through the dialyzer, and hypoglycemia can occur within 2–3 h after hemodialysis initiation (15). Hypoglycemia may stimulate the secretion of counter-regulatory hormones, such as glucagon, epinephrine, and cortisol, leading to rebound hyperglycemia after dialysis (36, 37). Excess glucose fluctuations can alter cardiovascular function. Hypoglycemia can stimulate catecholamines release, leading to increased myocardial contractility, myocardial workload, and oxygen consumption (37). Hyperglycemia also results in increased oxidative stress, inflammation, cell death, and glucotoxicity effects, which results in endothelial dysfunction and affects the microvasculature (38).

A recent study suggested a treatment paradigm shift from the triad goal, including fasting glucose, postprandial glucose, and HbA1c, to the pentad goal, where glycemic variability and quality of life are added to the abovementioned triad (39). In particular, a more flexible treatment strategy is required for glycemic control because of the changes in glucose and insulin metabolism caused by various factors in patients with diabetes undergoing hemodialysis.

In addition, several studies have reported that blood glucose control using CGM was cost-effective than SMBG, although the medical cost system and health insurance system differ from country to country (40–44). It has been associated with reduced insulin use, lower incidence of complications, increased life expectancy, and improved quality-of-life. CGM can be a tool of a cost-effective disease management option.

Our study had some limitations. First, it was a single-center study with a small number of patients. Second, a comparison rebound with SMBG during the study period would have been more helpful for identifying the usefulness of CGM as a tool for improving glycemic control. Nevertheless, several studies have reported a strong correlation between SMBG and CGM (20–23). Our center offers the “Education program for all of diabetes” for first-time diagnosed diabetes patients. This program contains information about SMBG and writing a diary. The mean diabetes duration among patients participating in this study was 22.9 ± 7.0 years, and the shortest duration was 7.5 years. Therefore, all patients who participated in this study performed SMBG and writing a glucose diary regularly as usual before enrollment in this study. Based on this, it may be said that the mean glucose levels, HbA1c levels, and CGM metrics of the baseline period (T0) indirectly showed the results of the SMBG-based treatment. In addition, since SMBG and writing a glucose diary were not new monitoring methods added during the study, they would have had little effect on the positive results. Finally, as hypoglycemia occurred relatively rarely in the present study, it was difficult to determine whether CGM could significantly reduce hypoglycemia. Instead, uncontrolled hyperglycemia was improved without an increase in hypoglycemia events, resulting in improvements in glycemic variability.

To the best of our knowledge, only a few CGM-based treatment intervention studies have been conducted in patients undergoing hemodialysis. In the first study, 28 patients with diabetes on hemodialysis showed significant improvement in the HbA1c levels from 8.4 to 7.6% after 3 months (5). In that study, patients were monitored with CGM for 54 h; however, in our study, patients were monitored with CGM for 7 days. In the second study, 15 patients with diabetes on hemodialysis had glucose control based on SMBG for 6 weeks, followed by CGM-based management for another 6 weeks (6). They showed that tighter blood glucose control was achieved, without an increase in hypoglycemia, using CGM rather than SMBG. In the present study, the difference in blood glucose patterns between the hemodialysis-on and hemodialysis-off days was analyzed, and this allowed individualized patient treatment. Above all, in the present study, our center’s central dialysis fluid delivery system could reduce bias affecting the blood glucose levels by supplying dialysate with the same glucose concentration to all patients.

## 5. Conclusion

The present study suggested that CGM could be a promising tool for individualizing treatment strategies for glycemic variability and improving blood glucose levels in patients with diabetes undergoing hemodialysis, in whom glycemic control is complex and difficult. Larger scale and longer studies are needed to confirm the usefulness of CGM for improving clinical outcomes, and mortality in patients with ESKD.

## Data availability statement

The raw data supporting the conclusions of this article will be made available by the authors, without undue reservation.

## Ethics statement

This study was approved by the Ethics Committee of the Eulji University School of Medicine in South Korea (IRB No. EMC 2021-07-013-001) and was performed in accordance with the principle of Declaration of Helsinki. All study participants provided written informed consent prior to enrollment in the study.

## Author contributions

SL, SYL, KMK, and JHS: conception, design, conduct of the study and the analysis, and interpretation of the results. SL: writing the first draft of the manuscript. All the authors edited, reviewed, and approved the final version of the manuscript.

## References

[1] Johansen K Chertow G Gilbertson D Herzog C Ishani A Israni A US renal data system 2021 annual data report: epidemiology of kidney disease in the United States. *Am J Kidney Dis.* (2022) 79:A8–12. 35331382 10.1053/j.ajkd.2022.02.001 PMC8935019

[2] Choi H Han K Oh T Suh S Kim M Kim C Trends in the incidence and prevalence of end-stage renal disease with hemodialysis in entire Korean population: a nationwide population-based study. *Medicine.* (2021) 100:e25293. 10.1097/MD.0000000000025293 33787616 PMC8021352

[3] Lee M Ha K Kim D Park I. Trends in the incidence, prevalence, and mortality of end-stage kidney disease in South Korea. *Diabetes Metab J.* (2020) 44:933–7. 10.4093/dmj.2020.0156 33389960 PMC7801765

[4] Sun B Luo Z Zhou J. Comprehensive elaboration of glycemic variability in diabetic macrovascular and microvascular complications. *Cardiovasc Diabetol.* (2021) 20:9. 10.1186/s12933-020-01200-7 33413392 PMC7792304

[5] Képénékian L Smagala A Meyer L Imhoff O Alenabi F Serb L Continuous glucose monitoring in hemodialyzed patients with type 2 diabetes: a multicenter pilot study. *Clin Nephrol.* (2014) 82:240–6. 10.5414/CN108280 25161114

[6] Joubert M Fourmy C Henri P Ficheux M Lobbedez T Reznik Y. Effectiveness of continuous glucose monitoring in dialysis patients with diabetes: the DIALYDIAB pilot study. *Diabetes Res Clin Pract.* (2015) 107:348–54. 10.1016/j.diabres.2015.01.026 25638452

[7] Mak R DeFronzo R. Glucose and insulin metabolism in uremia. *Nephron.* (1992) 61:377–82. 10.1159/000186953 1501732

[8] Shurraw S Hemmelgarn B Lin M Majumdar S Klarenbach S Manns B Association between glycemic control and adverse outcomes in people with diabetes mellitus and chronic kidney disease: a population-based cohort study. *Arch Intern Med.* (2011) 171:1920–7. 10.1001/archinternmed.2011.537 22123800

[9] McMurray S Johnson G Davis S McDougall K. Diabetes education and care management significantly improve patient outcomes in the dialysis unit. *Am J Kidney Dis.* (2002) 40:566–75. 10.1053/ajkd.2002.34915 12200809

[10] Bomholt T Adrian T Nørgaard K Ranjan A Almdal T Larsson A The Use of HbA1c, glycated albumin and continuous glucose monitoring to assess glucose control in the chronic kidney disease population including dialysis. *Nephron.* (2021) 145:14–9. 10.1159/000511614 33264783

[11] Speeckaert M Van Biesen W Delanghe J Slingerland R Wiecek A Heaf J Are there better alternatives than haemoglobin A1c to estimate glycaemic control in the chronic kidney disease population? *Nephrol Dial Transplant.* (2014) 29:2167–77. 10.1093/ndt/gfu006 24470517

[12] Monnier L Colette C Owens D. Glycemic variability: the third component of the dysglycemia in diabetes. Is it important? How to measure it. *J Diabetes Sci Technol.* (2008) 2:1094–100. 10.1177/193229680800200618 19885298 PMC2769808

[13] Nalysnyk L Hernandez-Medina M Krishnarajah G. Glycaemic variability and complications in patients with diabetes mellitus: evidence from a systematic review of the literature. *Diabetes Obes Metab.* (2010) 12:288–98. 10.1111/j.1463-1326.2009.01160.x 20380649

[14] Draznin B Aroda V Bakris G Benson G Brown F Freeman R 6. Glycemic targets: standards of medical care in diabetes-2022. *Diabetes Care.* (2022) 45:S83–96. 10.2337/dc22-S006 34964868

[15] Gai M Merlo I Dellepiane S Cantaluppi V Leonardi G Fop F Glycemic pattern in diabetic patients on hemodialysis: continuous glucose monitoring (CGM) analysis. *Blood Purif.* (2014) 38:68–73. 10.1159/000362863 25300368

[16] Welsh J Kaufman F Lee S. Accuracy of the Sof-sensor glucose sensor with the iPro calibration algorithm. *J Diabetes Sci Technol.* (2012) 6:475–6. 10.1177/193229681200600237 22538161 PMC3380794

[17] Battelino T Danne T Bergenstal R Amiel S Beck R Biester T Clinical targets for continuous glucose monitoring data interpretation: recommendations from the international consensus on time in range. *Diabetes Care.* (2019) 42:1593–603. 10.2337/dci19-0028 31177185 PMC6973648

[18] Zelnick L Batacchi Z Ahmad I Dighe A Little R Trence D Continuous glucose monitoring and use of alternative markers to assess glycemia in chronic kidney disease. *Diabetes Care.* (2020) 43:2379–87. 10.2337/dc20-0915 32788282 PMC7510019

[19] Maiorino M Signoriello S Maio A Chiodini P Bellastella G Scappaticcio L Effects of continuous glucose monitoring on metrics of glycemic control in diabetes: a systematic review with meta-analysis of randomized controlled trials. *Diabetes Care.* (2020) 43:1146–56. 10.2337/dc19-1459 32312858

[20] Divani M Georgianos P Didangelos T Iliadis F Makedou A Hatzitolios A Comparison of glycemic markers in chronic hemodialysis using continuous glucose monitoring. *Am J Nephrol.* (2018) 47:21–9. 10.1159/000485843 29275415

[21] Riveline J Teynie J Belmouaz S Franc S Dardari D Bauwens M Glycaemic control in type 2 diabetic patients on chronic haemodialysis: use of a continuous glucose monitoring system. *Nephrol Dial Transplant.* (2009) 24:2866–71. 19389864 10.1093/ndt/gfp181

[22] Jung H Kim H Kim M Yoon J Ahn H Cho Y Analysis of hemodialysis-associated hypoglycemia in patients with type 2 diabetes using a continuous glucose monitoring system. *Diabetes Technol Ther.* (2010) 12:801–7. 10.1089/dia.2010.0067 20809681

[23] Qayyum A Chowdhury T Oei E Fan S. Use of continuous glucose monitoring in patients with diabetes mellitus on peritoneal dialysis: correlation with glycated hemoglobin and detection of high incidence of unaware hypoglycemia. *Blood Purif.* (2016) 41:18–24. 10.1159/000439242 26960210

[24] Beck R Bergenstal R Cheng P Kollman C Carlson A Johnson M The relationships between time in range, hyperglycemia metrics, and HbA1c. *J Diabetes Sci Technol.* (2019) 13:614–26. 10.1177/1932296818822496 30636519 PMC6610606

[25] Vigersky R McMahon C. The relationship of hemoglobin A1C to time-in-range in patients with diabetes. *Diabetes Technol Ther.* (2019) 21:81–5. 10.1089/dia.2018.0310 30575414

[26] Patel A MacMahon S Chalmers J Neal B Billot L Woodward M Intensive blood glucose control and vascular outcomes in patients with type 2 diabetes. *N Engl J Med.* (2008) 358:2560–72. 10.1056/NEJMoa0802987 18539916

[27] Colette C Monnier L. Acute glucose fluctuations and chronic sustained hyperglycemia as risk factors for cardiovascular diseases in patients with type 2 diabetes. *Horm Metab Res.* (2007) 39:683–6. 10.1055/s-2007-985157 17846977

[28] Lu J Ma X Zhou J Zhang L Mo Y Ying L Association of time in range, as assessed by continuous glucose monitoring, with diabetic retinopathy in type 2 diabetes. *Diabetes Care.* (2018) 41:2370–6. 10.2337/dc18-1131 30201847

[29] Dhatariya K Li Ping Wah-Pun Sin E Cheng J Li F Yue A Gooday C The impact of glycaemic variability on wound healing in the diabetic foot - A retrospective study of new ulcers presenting to a specialist multidisciplinary foot clinic. *Diabetes Res Clin Pract.* (2018) 135:23–9. 10.1016/j.diabres.2017.10.022 29097286

[30] Li T Yang C Tseng S Li C Liu C Lin W Visit-to-visit variations in fasting plasma glucose and hba1c associated with an increased risk of Alzheimer disease: Taiwan diabetes study. *Diabetes Care.* (2017) 40:1210–7. 10.2337/dc16-2238 28705834

[31] Chiu H Li T Li C Liu C Lin W Lin C. Visit-to-visit glycemic variability is a strong predictor of chronic obstructive pulmonary disease in patients with type 2 diabetes mellitus: competing risk analysis using a national cohort from the Taiwan diabetes study. *PLoS One.* (2017) 12:e0177184. 10.1371/journal.pone.0177184 28489885 PMC5425194

[32] Xu F Zhao L Su J Chen T Wang X Chen J The relationship between glycemic variability and diabetic peripheral neuropathy in type 2 diabetes with well-controlled HbA1c. *Diabetol Metab Syndr.* (2014) 6:139. 10.1186/1758-5996-6-139 25530811 PMC4272789

[33] Monnier L Mas E Ginet C Michel F Villon L Cristol J Activation of oxidative stress by acute glucose fluctuations compared with sustained chronic hyperglycemia in patients with type 2 diabetes. *JAMA.* (2006) 295:1681–7. 10.1001/jama.295.14.1681 16609090

[34] Matsutani D Sakamoto M Iuchi H Minato S Suzuki H Kayama Y Glycemic variability in continuous glucose monitoring is inversely associated with baroreflex sensitivity in type 2 diabetes: a preliminary report. *Cardiovasc Diabetol.* (2018) 17:36. 10.1186/s12933-018-0683-2 29514695 PMC5840775

[35] Kazempour-Ardebili S Lecamwasam V Dassanyake T Frankel A Tam F Dornhorst A Assessing glycemic control in maintenance hemodialysis patients with type 2 diabetes. *Diabetes Care.* (2009) 32:1137–42. 10.2337/dc08-1688 19196889 PMC2699727

[36] Spoto B Pisano A Zoccali C. Insulin resistance in chronic kidney disease: a systematic review. *Am J Physiol Renal Physiol.* (2016) 311:F1087–108. 10.1152/ajprenal.00340.2016 27707707

[37] Desouza C Bolli G Fonseca V. Hypoglycemia, diabetes, and cardiovascular events. *Diabetes Care.* (2010) 33:1389–94. 10.2337/dc09-2082 20508232 PMC2875462

[38] Mapanga R Essop M. Damaging effects of hyperglycemia on cardiovascular function: spotlight on glucose metabolic pathways. *Am J Physiol Heart Circ Physiol.* (2016) 310:H153–73. 10.1152/ajpheart.00206.2015 26519027

[39] Glycemic Pentad Forum. Glycemic pentad. *J Assoc Physicians India.* (2017) 65:68–79. 28792171

[40] Huang E O’Grady M Basu A Winn A John P Lee J The cost-effectiveness of continuous glucose monitoring in type 1 diabetes. *Diabetes Care.* (2010) 33:1269–74. 10.2337/dc09-2042 20332354 PMC2875436

[41] Fonda S Graham C Munakata J Powers J Price D Vigersky R. The cost-effectiveness of real-time continuous glucose monitoring (RT-CGM) in type 2 diabetes. *J Diabetes Sci Technol.* (2016) 10:898–904. 10.1177/1932296816628547 26843480 PMC4928220

[42] Rotondi M Wong O Riddell M Perkins B. Population-level impact and cost-effectiveness of continuous glucose monitoring and intermittently scanned continuous glucose monitoring technologies for adults with type 1 diabetes in canada: a modeling study. *Diabetes Care.* (2022) 45:2012–9. 10.2337/dc21-2341 35834175 PMC9472499

[43] Tsuji S Ishikawa T Morii Y Zhang H Suzuki T Tanikawa T Cost-effectiveness of a continuous glucose monitoring mobile app for patients with type 2 diabetes mellitus: analysis simulation. *J Med Internet Res.* (2020) 22:e16053. 10.2196/16053 32940613 PMC7530685

[44] Wan W Skandari M Minc A Nathan A Winn A Zarei P Cost-effectiveness of continuous glucose monitoring for adults with type 1 diabetes compared with self-monitoring of blood glucose: the DIAMOND randomized trial. *Diabetes Care.* (2018) 41:1227–34. 10.2337/dc17-1821 29650803 PMC5961392

