# Supplementary material for: Usefulness of continuous glucose monitoring of blood glucose control in patients with diabetes undergoing hemodialysis: A pilot study
Source: Front Med (Lausanne). 2023 Apr 6;10:1145470. doi: 10.3389/fmed.2023.1145470 (PMC10117913; doi:10.3389/fmed.2023.1145470)
Supplement: Supplementary file 1 [file Table_1.docx]

**Supplementary Data**

**Supplementary Table 1. Treatment intervention and changes in CGM metrics for each of all patients**

| **Patient** | **T0** | | | | **T1** | | | | **Treatment intervention** |
| --- | --- | --- | --- | --- | --- | --- | --- | --- | --- |
|  | **HbA1c** | **TIR** | **SD** | **%CV** | **HbA1c** | **TIR** | **SD** | **%CV** |  |
| Number  HD-on  HD-off | % | % of readings | mg/dL | % | % | % of readings | mg/dL | % |  |
| 1 | 9.2 |  |  |  | 7.2 |  |  |  |  |
|  |  | 36 | 29 | 21.2 |  | 59 | 44 | 26.5 |  |
|  |  | 25 | 62 | 28 |  | 40 | 38 | 19 |  |
| 2 | 5.4 |  |  |  | 5.2 |  |  |  | No intervention |
|  |  | 100 | 12 | 14 |  | 100 | 17 | 14 |  |
|  |  | 97 | 20 | 14 |  | 97 | 18 | 14 |  |
| 3 | 7.1 |  |  |  | 7.4 |  |  |  | Insulin degludec injection on the morning  → the evening |
|  |  | 47 | 82 | 39.4 |  | 67 | 48 | 31.1 |  |
|  |  | 35 | 82 | 39 |  | 50 | 48 | 32 |  |
| 4 | 7.3 |  |  |  | 6.0 |  |  |  | Administered mixed insulin aspart protamine  1 hour earlier |
|  |  | 33 | 48 | 30.5 |  | 28 | 34 | 27 |  |
|  |  | 47 | 42 | 22 |  | 67 | 34 | 22 |  |
| 5 | 7.0 |  |  |  | 6.8 |  |  |  | Encourage exercise after meals |
|  |  | 73 | 28 | 17 |  | 79 | 17 | 18.6 |  |
|  |  | 72 | 35 | 22 |  | 86 | 30 | 21 |  |
| 6 | 8.2 |  |  |  | 7.7 |  |  |  | Insulin glargine  4 units ↑ |
|  |  | 26 | 69 | 36 |  | 63 | 48 | 31 |  |
|  |  | 26 | 62 | 36 |  | 63 | 44 | 31 |  |
| 7 | 5.8 |  |  |  | 5.5 |  |  |  | No intervention |
|  |  | 100 | 16 | 13 |  | 100 | 16 | 15 |  |
|  |  | 99 | 17 | 14 |  | 99 | 16 | 12 |  |
| 8 | 7.7 |  |  |  | 8.1 |  |  |  | Insulin aspart 2 units ↓on the morning of dialysis day |
|  |  | 88 | 34 | 26 |  | 85 | 34 | 25.5 |  |
|  |  | 88 | 37 | 27 |  | 80 | 26 | 26 |  |
| 9 | 8.5 |  |  |  | 7.7 |  |  |  | Education on regular meals |
|  |  | 62 | 53 | 34.6 |  | 60 | 52 | 27.6 |  |
|  |  | 62 | 48 | 35 |  | 60 | 52 | 27 |  |
| 10 | 6.0 |  |  |  | 6.5 |  |  |  | Insulin glargine  4 units ↑ |
|  |  | 43 | 70 | 26 |  | 56 | 49 | 22 |  |
|  |  | 39 | 83 | 35 |  | 62 | 48 | 26 |  |
| 11 | 6.6 |  |  |  | 6.3 |  |  |  | Gliclazide MR 60 mg → Gliclazide MR 30 mg and linagliptin  5 mg |
|  |  | 82 | 29 | 21 |  | 82 | 29 | 20 |  |
|  |  | 85 | 35 | 26 |  | 85 | 40 | 28 |  |
| 12 | 7.5 |  |  |  | 5.0 |  |  |  | Insulin detemir → Insulin degludec |
|  |  | 62 | 45 | 26 |  | 58 | 43 | 26 |  |
|  |  | 48 | 47 | 24 |  | 92 | 34 | 23 |  |
| 13 | 9.7 |  |  |  | 9.4 |  |  |  | Mixed insulin aspart protamine  4-4 units ↑ |
|  |  | 16 | 72 | 22 |  | 58 | 58 | 20 |  |
|  |  | 11 | 61 | 24 |  | 65 | 44 | 20 |  |
| 14 | 6.0 |  |  |  | 5.5 |  |  |  | Add linagliptin 5 mg on the HD-off day |
|  |  | 74 | 48 | 36 |  | 76 | 39 | 35 |  |
|  |  | 77 | 47 | 35 |  | 77 | 39 | 32 |  |
| 15 | 8.8 |  |  |  | 7.4 |  |  |  | Add daily insulin degludec  10 units |
|  |  | 65 | 37 | 25.4 |  | 70 | 25 | 20 |  |
|  |  | 64 | 52 | 28 |  | 75 | 25 | 18 |  |
| 16 | 6.9 |  |  |  | 7.3 |  |  |  | Add gliclazide MR 30 mg after dialysis |
|  |  | 43 | 29 | 29 |  | 79 | 24 | 16 |  |
|  |  | 55 | 43 | 25 |  | 85 | 27 | 18 |  |
| 17 | 8.8 |  |  |  | 7.8 |  |  |  | Add daily insulin degludec  14 units |
|  |  | 32 | 44 | 23 |  | 65 | 43 | 25 |  |
|  |  | 26 | 39 | 17 |  | 63 | 40 | 24 |  |
| 18 | 7.3 |  |  |  | 6.8 |  |  |  | Gliclazide 120 mg → 60 mg on the HD-on day  Add Gemigliptin 50 mg on the HD-off day |
|  |  | 57 | 68 | 37 |  | 58 | 34 | 20 |  |
|  |  | 32 | 64 | 26 |  | 63 | 40 | 25 |  |

**CGM, continuous glucose monitoring; HbA1c, glycated hemoglobin A1c; TIR, time in range; SD, standard deviation; %CV, % coefficient of variation; HD, hemodialysis; MR, modified release.**
